# Compressively sampling the optical transmission matrix of a multimode fibre

**DOI:** 10.1038/s41377-021-00514-9

**Published:** 2021-04-21

**Authors:** Shuhui Li, Charles Saunders, Daniel J. Lum, John Murray-Bruce, Vivek K Goyal, Tomáš Čižmár, David B. Phillips

**Affiliations:** 1 School of Physics and Astronomy, University of Exeter, Exeter, EX4 4QL UK; 2 Wuhan National Laboratory for Optoelectronics, School of Optical and Electronic Information, Huazhong University of Science and Technology, Wuhan, 430074 Hubei China; 3 Department of Electrical and Computer Engineering, Boston University, Boston, MA 02215 USA; 4 Department of Physics and Astronomy, University of Rochester, 500 Wilson Blvd, Rochester, NY 14618 USA; 5 Department of Computer Science and Engineering, University of South Florida, Tampa, FL 33620 USA; 6 Leibniz Institute of Photonic Technology, Albert-Einstein-Straße 9, 07745 Jena, Germany; 7 Institute of Scientific Instruments of CAS, Královopolská 147, 612 64 Brno, Czech Republic

**Keywords:** Adaptive optics, Fibre optics and optical communications, Imaging and sensing, Micro-optics

## Abstract

The measurement of the optical transmission matrix (TM) of an opaque material is an advanced form of space-variant aberration correction. Beyond imaging, TM-based methods are emerging in a range of fields, including optical communications, micro-manipulation, and computing. In many cases, the TM is very sensitive to perturbations in the configuration of the scattering medium it represents. Therefore, applications often require an up-to-the-minute characterisation of the fragile TM, typically entailing hundreds to thousands of probe measurements. Here, we explore how these measurement requirements can be relaxed using the framework of compressive sensing, in which the incorporation of prior information enables accurate estimation from fewer measurements than the dimensionality of the TM we aim to reconstruct. Examples of such priors include knowledge of a memory effect linking the input and output fields, an approximate model of the optical system, or a recent but degraded TM measurement. We demonstrate this concept by reconstructing the full-size TM of a multimode fibre supporting 754 modes at compression ratios down to ∼5% with good fidelity. We show that in this case, imaging is still possible using TMs reconstructed at compression ratios down to ∼1% (eight probe measurements). This compressive TM sampling strategy is quite general and may be applied to a variety of other scattering samples, including diffusers, thin layers of tissue, fibre optics of any refractive profile, and reflections from opaque walls. These approaches offer a route towards the measurement of high-dimensional TMs either quickly or with access to limited numbers of measurements.

## Introduction

The scattering of light was long thought to be an insurmountable barrier preventing imaging through opaque materials. However, elastic scattering from static objects is deterministic, and in the last decade, it has been shown that it is possible to use wavefront shaping with spatial light modulators to characterise and subsequently cancel out complicated scattering effects^1–3^. Therefore, light that has undergone multiple scattering can be unscrambled to see through opaque media, such as frosted glass^4^, biological tissue^5,6^, or multimode optical fibres (MMFs)^7–9^.

The measurement of the transmission matrix (TM) of the scattering material in question is a powerful way to achieve this light control capability^10^. The TM can be understood as part of the optical response function of a scatterer: it is a linear operator relating a set of input ‘probe’ fields incident on one side of the scatterer to a new set of output fields leaving on the opposite side. Once the TM has been characterised, it encodes how any linear combination of probe fields will be scrambled and, more importantly, how to unscramble them again^11^. This versatile approach simplifies the task of ‘un-doing’ scattering effects, connecting the light fields on either side of a scatterer and thereby circumventing the need to consider the interaction of the light with the nano-scale structure of the scatterer itself^12,13^.

Beyond imaging, the information-rich nature of the high-dimensional TM is finding applications in a growing number of areas. Examples include the identification of the principal modes of MMFs to maximise spatial coherence for high-capacity telecom applications^14^, the optimisation of energy delivery inside scattering materials^15,16^, the design of optimised optical trapping fields through random scattering systems^17,18^, and the creation of new forms of all-optical classical^19^ and quantum^20^ information processing.

The output fields emerging from complicated scattering systems result from the interference of light that has taken many different optical paths through the scatterer. This multi-path interference typically renders high-dimensional TMs extremely sensitive to perturbations in the configurations of the systems they represent. Even recently recorded TM measurements tend to degrade over a period of time (e.g. minutes to hours depending on the stability of the scatterer in question and the optical system used to characterise it). To maintain high fidelity, the TM of a scattering system typically needs to be characterised regularly. The number of independent ‘pixels’ in an image that can be transmitted through a disordered medium is conventionally proportional to the number of linearly independent probe measurements which have been made during TM calibration—a number that can easily extend into the thousands. Therefore, establishing new ways to accelerate TM measurement is a useful step towards the deployment of TM-reliant technologies in real-world scenarios.

In this work, we explore how the number of probe measurements needed to characterise the TM of a scattering system can be reduced. In many cases, we have advance knowledge of some general characteristics of the TM we wish to measure. These priors may take various forms, including knowledge of the existence of a ‘memory effect’ giving rise to characteristic statistical relationships of the input and output fields^21–23^, access to a model approximating the optical system^24^, or a recent but degraded TM measurement of the same or a similar object. Here, we provide a guide to the incorporation of these priors into TM reconstruction using the framework of compressive sensing^25^. We experimentally validate this technique by using it to reconstruct the high-fidelity TM of an MMF supporting 754 spatial modes using only 38 measurements (∼5% of the fibre’s mode capacity). Furthermore, we show that TMs with sufficient fidelity for imaging can be reconstructed using as few as eight measurements (∼1% compression). These methods are universal and may be applied to a range of other scattering systems, including thin layers of tissue, optical diffusers, and scattering from opaque walls.

## Results

### Concept

The monochromatic complex-valued *N*-dimensional TM, **T** ∈ C^*N* × *N*^, describes how an incident field **a** ∈ C^*N*^ is transformed via propagation through a scatterer into an output field **b** ∈ C^*N*^, where **b** = **Ta**. Here, **a** and **b** are complex-valued column vectors representing the vectorised (reshaped) 2D input and output fields at a single wavelength and polarisation.

Experimentally, an unknown TM is often measured by injecting a sequence of orthogonal input probe fields, where the *n*th input is denoted by **a**_*n*_, and recording how each input field is transformed by propagation through the scatterer into the corresponding output field **b**_*n*_. The output field is typically measured with a camera, and off-axis digital holography with a coherent reference beam can be used to recover both its amplitude and phase from a single image^26^. The TM of the scatterer, **T**, can then be constructed from these measurements by assigning the *n*th output field **b**_*n*_ to the *n*th column of **T**^27^. In this construction, the basis in which **T** is represented is inherited from the bases in which the input and output fields are represented—but subsequently, we are at liberty to numerically transform its representation into any input and output bases of our choosing. From here on, we refer to this reconstruction technique as *columnwise* reconstruction. Evidently, the number of independent measurements, *m*, that we need to make should be equal to or greater than the number of orthogonal modes, *N*, that we wish to control—where here we have defined the recording of an entire output field, simultaneously in a single camera image, as an individual ‘measurement’.

Shifting our attention to an under-sampled case, let us consider the following situation: if we have prior knowledge of a basis in which the TM is perfectly diagonal, then we need only make a single measurement to recover all of the complex amplitudes of the elements on the diagonal. In this case, we inject a probe field **a**_1_ consisting of a known superposition of all of the modes represented in the diagonal basis, and at the output, we measure the transformed field **b**_1_. Our prior tells us that there has been no coupling between modes, and thus, we can numerically decompose **b**_1_ into the diagonal basis and find the complex diagonal elements of the TM by inspecting how the amplitude and phase of each mode have changed compared to the known input. This example illustrates how prior knowledge allows us to recover signals from far fewer measurements than the dimensionality of the signal. In this case, we can perform the absolute minimum number of measurements (i.e. one) because we have complete knowledge of both the sparsifying basis and the sparsity pattern (i.e. power is found only on the diagonal). Although our level of prior knowledge is often much weaker than that in this example, the field of compressive sensing^25^, and more generally, the concept of inference, provide the tools to make the best use of any priors we have available to reconstruct high-fidelity TMs with reduced numbers of measurements.

To proceed, we note that instead of using the columnwise method, we can construct a linear system of equations to which **t**, the vectorised form of **T**, is the solution:

1 $${\mathbf{St}} \,=\, {\mathbf{y}}$$

where **t** ∈ ${\mathrm{C}}^{{\mathrm{\it{N}}}^{2}}$ is a column vector containing the unknown complex elements of the TM, which may be represented in an arbitrary basis of our choosing; **S** ∈ ${{\mathrm{C}}^{{{{Nm}}}\times{{{N}}}^{2}}}$ is a ‘sensing’ matrix determined by the set of input modes used to probe the TM; and **y** ∈ C^*Nm*^ is a column vector representing the output measurements. The entries of the known matrix **S** and the vector **y** depend on our choice of the basis representation of **t**. See the ‘Methods’ for the details of how **S** and **y** are constructed from the set of known input and measured output fields.

If the TM is over-sampled (i.e. *m* > *N*) and **S** is of full rank, then **t** may be found by solving Eq. 1 using standard methods that minimise an error term *η* given by the square of the Euclidean norm of the residual: $\eta \,=\, ||{\mathbf{St}} \,-\, {\mathbf{y}}||_2^2$, which accounts for any inconsistencies in Eq. 1 due to noise in the measurements. If the TM is critically sampled (i.e. *m* = *N*) and **S** is again of full rank, then **t** may be found through direct inversion. However, if the TM is under-sampled (i.e. *m* < *N*), then **S** is rank-deficient and Eq. 1 has an infinite number of possible solutions, only one of which represents the true TM. Here, our task is to use any prior knowledge of the system we may have to constrain the possible solutions to Eq. 1 and locate a solution close to the correct one. We note that this prior knowledge could also be used to counteract measurement noise in the over-sampled and critically sampled cases.

A strong prior is knowledge of a basis in which each input mode does not scatter into many output modes, meaning that the TM is sparse. An even stronger prior is advance knowledge of which output modes each input is likely to scatter into. When might we have access to such priors regarding the TM of a scattering object? There are several situations that provide information of this sort. First, it has recently been highlighted that if a scattering system is known to possess a memory effect, then this is equivalent to knowledge of a basis in which the TM of the scatterer is quasi-diagonal—meaning that a significant proportion of the power is found on the main diagonal^22,23^. Second, if we have access to a model approximating the optical system in question, then we can use this to find a quasi-diagonalizing basis by simulating the TM and diagonalising it. Third, if we have performed a recent but degraded TM measurement on the same scatterer, then this can also be diagonalised to find a sparse basis. We emphasise that the situation we consider in this article is when we have advance knowledge of the general characteristics of the TM we wish to find, but none of the above alone reveals sufficient information to build an accurate TM—consequently, we still need to make some probe measurements. Our aim is to use the available prior information, along with a small number of new measurements, to reconstruct an accurate TM for the scatterer in question.

### Compressively sampling the TM of an MMF

We now consider the example of a MMF. The control of light fields through MMFs has recently attracted growing attention, as MMF-based micro-endoscopy promises video-rate imaging with sub-cellular resolution deep within tissue at the tip of a needle^28–30^. MMFs have also been used as mixing elements for classical and quantum optical computing schemes^19,20^. Modal dispersion means that an image projected onto one end of an MMF is scrambled into a speckle pattern at the other end, and so, before an MMF can be deployed as a micro-endoscope, it is necessary to first characterise its TM to understand how to invert this scrambling process^7–9^. Unfortunately, any slight bending deformations or temperature fluctuations modify the TM and thus cause the imaging performance of current fibre technology to quickly degrade^31^. Therefore, in the context of emerging MMF-based clinical imaging scenarios, these stability constraints mean that the TMs of MMFs may need to be regularly characterised.

The approximate cylindrical symmetry of an MMF tells us much about the structure of the TM in advance of its measurement. Solving the monochromatic wave equation in an idealised straight section of a step-index fibre reveals a set of orthogonal circularly polarised eigenmodes, known as propagation-invariant modes (PIMs)^24^. The PIMs maintain a constant spatial profile and polarisation during propagation. This means that in the ideal case, power does not couple between these eigenmodes, and the TM in the PIM basis is unitary and perfectly diagonal. This implies that ideal fibres have a 2π rotational memory effect^32^ and a quasi-radial memory effect that reaches over the entire output facet^23^. Although real optical fibres differ from this idealised case, Plöschner et al. recently showed that the TM of a short length of step-index MMF is relatively sparse and strongly diagonal when represented in the PIM basis and that coupling between the left- and right-handed circular polarisations is minimal^24^. Details of the PIMs and how they are calculated are given, for example, in refs. ^23,24^.

**Fig. 1:**
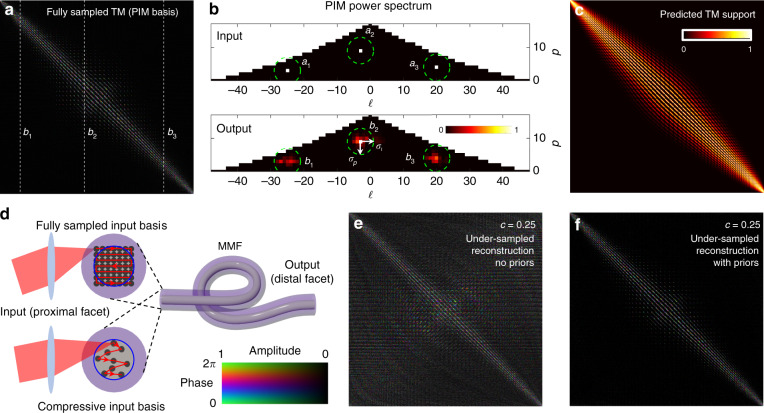
Compressive sampling of the TM of an MMF. **a** A fully sampled TM of an ~30 cm segment of step-index MMF supporting 754 modes at a wavelength of 633 nm (NA = 0.22, core diameter = 50 μm). Here, represented in the PIM basis, the TM shows strong diagonal features and a well-defined off-diagonal structure. **b** PIMs are indexed by an azimuthal index $\ell$, describing the orbital angular momentum carried by a PIM, and a radial index *p*, which is related to the degree of radial momentum carried by the PIM. The heat maps in (**b**) show the power spectra of three input fibre modes (a_1_, a_2_, and a_3_) and the three corresponding output fields (b_1_, b_2_, and b_3_) represented in the ($\ell$, *p*)-space of the PIM basis, which also correspond to the three columns marked in (**a**). The colour of pixel ($\ell$, *p*) corresponds to the relative power found in the mode with indices ($\ell$, *p*). We see that each input couples only to modes with similar $\ell$ and *p* indices during propagation through the fibre (see also Movie 1 and SI Fig. S7). **c** A support for the TM can be predicted by estimating the degrees of modal coupling $\sigma_{\ell}$ and $\sigma_p$, indicating areas that are likely to contain little to no power (see the Methods for details); here, $\sigma_{\ell}$ = 4 and $\sigma_p$ = 2. **d** Schematic showing the input modes (a focussed point swept across the input facet) used to fully sample (top) or under-sample (bottom) the TM of the MMF. In the over-sampled case, the input focussed beam is scanned across an overlapping Cartesian grid of points. In the under-sampled case, points are addressed in a hyper-uniform pattern. **e** An under-sampled TM (*c* = 0.25) reconstructed using the columnwise method with no priors and then transformed into the PIM basis. In this case, the TM is evidently reconstructed with low fidelity: it exhibits considerable off-diagonal power, and its normalised correlation with the fully sampled TM is 0.23. This correlation is calculated as the squared modulus of the normalised overlap integral between the complex-valued reconstructed TM and the fully sampled TM. **f** TM reconstructed using the same data as in (**e**) but using FISTA with a predicted support. In this case, the TM is reconstructed with higher fidelity, and the normalised correlation with the fully sampled TM rises to 0.88. Note: (**a**) and (**f**) are reproduced on a larger scale in Supplementary Information (SI) Figs. S3, S4

Figure 1a shows an example of an experimentally measured fully sampled TM of a ~30 cm strand of step-index MMF, represented in the circularly polarised PIM basis. The TM was recorded at a wavelength of 633 nm, at which the fibre supports *N* = 754 modes per polarisation. We note that this measurement was conducted for a single input and output circular polarisation and that the manufacturer’s quoted mean values of core diameter = 50 μm and NA = 0.22 were used to calculate the transformation to the PIM basis. In our experiments, we have typically found that ∼10% of the power is on the main diagonal and that the power is concentrated into relatively few elements, meaning that the TM is sparse, as anticipated by our model. In general, mode coupling increases with fibre length, and accordingly, the degree of mode coupling in the TM can be quantified by the ratio *L*/*l*_f_, where *L* is the fibre length and *l*_f_ is the effective transport mean free path in the fibre mode (PIM) basis, i.e. *l*_f_ is the fibre length beyond which modal coupling is maximised and the TM can be considered fully coupled^33^. In this case, our experimentally measured TM corresponds to *L*/*l*_f_ ∼ 0.02. See the ‘Methods’ for the details of how *l*_f_ is numerically estimated.

Intriguingly, the power in the TM spreads away from the diagonal in a well-structured manner, suggesting that we should be able to derive an estimate of the sparsity pattern. The root of this structure is revealed by considering how the PIMs couple preferentially to others of similar azimuthal (${\ell}$) and radial (*p*) mode indices when they are distorted by a small amount. For example, the experimentally measured coupling of three input fibre modes that have undergone propagation through our fibre is shown in power spectrum plots in Fig. 1b, where we see that the power is coupled only locally in this representation. SI Movie 1 displays the experimentally measured power coupling of every input PIM (see also the description in SI Fig. S7). Evidently, this shows that we can directly probe the transformation of multiple input PIMs simultaneously from a single probe measurement and in a single output camera frame, as long as the inputs have well-separated mode indices. Using the example shown in Fig. 1b, the transformation experienced by each of the three input modes can be separately measured at the output by transforming the field into the PIM basis and associating each ‘island’ of power with each individual input PIM.

To make use of our local coupling prior, we can model the coupling of PIMs as a 2D Gaussian function with standard deviations $\sigma_{\ell}$ and *σ*_p_ describing the degree of power overspill into adjacent modes. This enables the prediction of a map capturing the off-diagonal structure that we expect to observe in the TM, i.e. an estimate of the amplitude of the TM, as shown in Fig. 1c. This information can be used as an estimated ‘support’ to guide the reconstruction of the TM. The prediction of this support is parameterised by estimation of only two numbers ($\sigma_{\ell}$ and *σ*_p_). Therefore, for short lengths of MMF (up to tens of centimetres^24^) with a known core diameter and numerical aperture, both the sparsifying PIM basis (i.e. the transformation matrix from real space to the PIM space) and an estimate of the support are known in advance of any measurements and can be used in the reconstruction of the TM. We also note that through the judicious selection of the first few probe measurements, reasonably accurate estimates of $\sigma_{\ell}$ and *σ*_p_ can be found. For example, by injecting individual PIMs for the first few measurements, the mode coupling of these can be directly measured, which can then be used to estimate $\sigma_{\ell}$ and *σ*_p_ to predict the shape of the support. As shown in Fig. 1b, a single measurement of this kind is enough to estimate the support, although more measurements will lead to greater accuracy.

We are now equipped with strong priors regarding the TM of the MMF in advance of its measurement. So, what measurements should we make? As illustrated by our example at the start of the Concept section, a good measurement basis is incoherent with respect to the predicted sparse basis, i.e. each of our reduced number of probe measurements should excite many PIMs^25^. Ideally, all measurements should also be orthogonal to one another to ensure that each new measurement yields independent information about the scatterer. To satisfy these requirements, we perform measurements in a basis formed by a single diffraction-limited spot that can be focussed onto different locations across the core of the input facet of the MMF. Each of these foci excites many PIMs and thus has a high level of incoherence with the sparse TM basis (see SI Fig. S1). The spot locations are drawn from a disordered hyper-uniform array, which ensures that they do not overlap, and thus, the inputs are orthogonal. An example is shown in Fig. 1d, and further details of how this array was designed are given in the ‘Methods’. This probing basis also has the advantage of being experimentally straightforward to accurately create.

To reconstruct the full TM from our under-sampled measurement set, we incorporate our priors by solving the following optimisation problem:

2 $${\hat{\mathbf t}} \,=\, \mathop {{{\mathrm{arg}}\,{\mathrm{min}}}}\limits_{\mathbf{t}} \frac{1}{2}\underbrace {||{\mathbf{St}} \,-\, {\mathbf{y||}}_2^2}_{{\mathrm{Data}}\,{\mathrm{fidelity}}} \,+\, \underbrace {\lambda \left( {{\mathbf{1}} \,-\, {\mathbf{w}}} \right)^{\mathrm{T}}|{\mathbf{t}}|}_{{\mathrm{Sparsity}}}$$

where ${\hat{\mathbf t}}$ ∈ ${\mathrm{C}}^{{{N}}^{2}}$ is the final solution and **t** ∈ ${\mathrm{C}}^{{{N}}^{2}}$ is the decision variable, both represented in the sparse PIM basis. Here, **1** ∈ ${\mathrm{C}}^{{{N}}^{2}}$ is a column vector of ones, and |**t**| is the magnitude of the complex-valued **t**. Equation 2 specifies that the solution should both agree with our under-sampled set of measurements (first term on the right-hand side) and be sparse, with low absolute values in regions dictated by our estimated support (second term on the right-hand side). We minimise the square of the Euclidean norm in the data fidelity term, as we expect the noise to be normally distributed. The column vector **w** ∈ ${\mathrm{R}}^{{{N}}^{2}}$ is the vectorised predicted support, with values between 0 and 1, determined a priori through the estimation of $\sigma_{\ell}$ and σ_p_, as described in the Methods. It promotes solutions with magnitudes that adhere more closely to our predicted TM structure. The scalar *λ* is a tuneable parameter that weights the relative importance of the fidelity and sparsity terms (see the ‘Methods’ for how the value of this parameter is chosen).

The problem defined in Eq. 2 can be solved using a range of methods. Here, we use the fast iterative soft-thresholding algorithm (FISTA)^34^, chosen because it is capable of rapidly solving relatively large-scale problems with low memory requirements. Psuedo-code describing the algorithm is shown in Table 1. More details on how this problem is solved are given in the Methods.

**Table 1:** Pseudo-code describing the algorithm used to solve Eq. 2.

**Algorithm 1** FISTA for solving Eq. 2
**Input:** Initial estimate **t**^0^ Measurement vector **y** Sensing matrix **S** Regularisation strength $\lambda \,\ge\, 0$ Estimated support vector **w** Step size $\alpha \,<\, 1/L$ ($L$ is the Lipschitz constant of the gradient of the cost function).
**Output:** ${\hat{\mathbf t}}$ 1. $\mu \,=\, 1$, ${\mathbf{x}}^0 \,=\, {\mathbf{t}}^0$ 2. **while** not converged **do** 3. ${\mathbf{x}}^{k \,+\, 1} \,=\, P_{\alpha \lambda {\mathbf{w}}}\left( {{\mathbf{t}}^k \,-\, \alpha \underbrace {\left( {{\mathbf{S}}^\dagger \left( {{\mathbf{St}}^k \,-\, {\mathbf{y}}} \right)} \right)}_{{\mathrm{Data}}\,{\mathrm{fidelity}}\,{\mathrm{gradient}}}} \right)$ 4. $\mu ^{k \,+\, 1} \,=\, \frac{1}{2}\left( {1 \,+\, \sqrt {4\left( {\mu ^k} \right)^2 \,+\, 1} } \right)$ 5. ${\mathbf{t}}^{k \,+\, 1} \,=\, {\mathbf{x}}^{k \,+\, 1} \,+\, \left( {\left( {\mu ^k \,-\, 1} \right)/\mu ^{k \,+\, 1}} \right)\left( {{\mathbf{x}}^{k \,+\, 1} \,-\, {\mathbf{x}}^k} \right)$ 6. **end while** 7. Return ${\hat{\mathbf t}} \,=\, {\mathbf{t}}^{k \,+\, 1}$ where $P_\tau \left( {\mathbf{z}} \right)_i \,=\, {\mathrm{max}}\left( {0,1 \,-\, {\mathbf{\tau }}_i/|{\mathbf{z}}_i|} \right){\mathbf{z}}_i$ gives the solution to the proximal operator for the sparsity regularisation term, with ${\mathbf{\tau }} \,=\, \alpha \lambda {\mathbf{w}}$. Here ${\mathbf{S}}^\dagger \left( {{\mathbf{St}}^k \,-\, {\mathbf{y}}} \right)$ is the data fidelity gradient.

We aim to use an under-sampled set of measurements to reconstruct the TM of the step-index MMF shown in Fig. 1a, with *L* ∼ 30 cm and *N* = 754 at a wavelength of 633 nm, as detailed earlier. These parameters were chosen to reflect those used in prototype MMF-based micro-endoscopes^30^. The mode capacity of the MMF means that the TM capturing a single input and output circular polarisation consists of 754^2^ = 568516 complex elements when represented in the PIM basis. Reconstructing this TM without exploiting the use of priors requires at least 754 sequentially recorded probe measurements.

Our experimental set-up is shown in SI Fig. S2. The set-up is similar to that in ref. ^23^ and is based on a Mach-Zehnder interferometer. In brief, light from the laser source is split into two beam paths. The signal arm of the interferometer contains the MMF to be characterised, along with a digital micro-mirror device (DMD) used to spatially modulate the complex field of the light injected into the MMF^35–38^. The reference arm directs light around the MMF to be used as a coherent reference. The output facet of the MMF is imaged onto a high-speed camera, where it interferes with light from the reference arm to form an interferogram enabling measurement of the amplitude and phase of the output field in a single camera frame using off-axis digital holography^26^.

Once the TM has been reconstructed, it can be used to create an arbitrary light field **d** at the distal end of the MMF (consisting of any linear combination of the PIMs) by calculating the required proximal field **c** = **T**^†^**d**, where we have assumed that the TM is unitary and thus **T**^−1^ = **T**^†^. Here (.)^†^ denotes the conjugate transpose operation. Scanning imaging is achieved by appropriately shaping the input field to sweep a focussed spot over the distal facet^9^. Reflectance or fluorescence images can be captured by measuring the total reflected/fluorescently excited intensity that is transmitted back to the proximal end and correlating this signal with each known distal spot location, turning the system into a micro-endoscope^30^.

To investigate the level of compression that is experimentally achievable, we probed the TM of the MMF multiple times, in each case reducing the number of measurements, *m*, drawn from the hyper-uniform input spot basis (see the Methods). The compression ratio *c* is given by *c* = *m*/*N*. For each data set, we compared the performance of three different TM reconstruction algorithms incorporating different levels of prior knowledge about the MMF:

(i) *No priors*—columnwise method of reconstructing the TM.
(ii) *Sparsity prior*—FISTA incorporating prior knowledge of the basis in which we estimate the TM to be sparse (i.e. the PIM basis) but no knowledge of which modes the input light is scattered into, i.e. no knowledge of the support, meaning that in this case, **w** = 0 everywhere.
(iii) *Sparsity prior and estimate of support*—FISTA incorporating prior knowledge of both a sparse basis and a TM amplitude support estimate that promotes a diagonal structure, an example of which is shown in Fig. 1c. In this case, **w** is computed from estimates of $\sigma_{\ell}$ and *σ*_p_ (see the ‘Methods’).

Figure 1e, f show under-sampled TM reconstructions in the PIM basis (*c* ∼ 0.25) when no prior information is used (Fig. 1e) and when both sparsity and support priors are incorporated (Fig. 1f). These can both be compared with the fully sampled TM shown in Fig. 1a. Without the leveraging of priors, the correlation between the under-sampled TM and the fully sampled TM is low. In fact, this correlation is directly proportional to the compression ratio (correlation = 0.23 ∼ c). The incorporation of priors into the reconstruction process significantly boosts the fidelity of the under-sampled TM (correlation of 0.88).

**Fig. 2:**
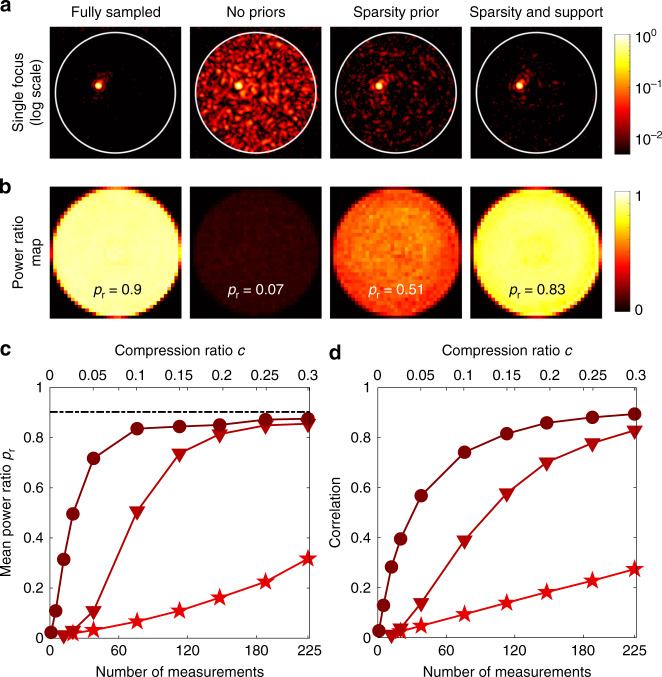
Compressively sampled TM fidelity. **a** Examples of output foci created using TMs reconstructed in different ways at a compression ratio of *c* = 0.1. **b** Corresponding power-ratio maps of the spots generated with each reconstruction strategy. **c** Graph showing how the mean power-ratio *p*_r_ varies as a function of *c* and the number of measurements, *m*, for three different reconstruction strategies incorporating different levels of prior knowledge. The black dash-dotted line corresponds to *p*_r_ = 0.9 in the fully sampled case. **d** Graph showing how the level of correlation between the under-sampled and fully sampled TMs varies as a function of *c*. Key for graphs (**c**, **d**): stars = no priors; triangles = sparsity prior; circles = sparsity and support priors, using the support shown in Fig. 1c

The fidelity of the reconstructed TMs can also be quantified by measuring how well they can be used to generate diffraction-limited foci at the output of the fibre. To do this, we calculated the mean power-ratio *p*_r_, defined as follows: for a Cartesian grid of points across the output facet, we calculated the ratio of the power within a small disk centred on the target focal position to the total power transmitted through the MMF. Figure 2a shows examples of diffraction-limited foci generated at the fibre output using TMs reconstructed with the different methods. *p*_r_ is the average power-ratio over all point positions across the core. Figure 2b shows the power-ratio map across the output facet in each example case.

We first benchmarked the fidelity of the fully sampled TM measurement with a high signal-to-noise ratio (SNR) by over-sampling the TM with an input basis consisting of a 41 × 41 Cartesian grid of points (see Fig. 1d). Therefore, *c* = (41)^2^/754 ∼ 2.2. The output foci generated using this over-sampled TM without the incorporation of priors yielded an experimental mean power-ratio of *p*_r_ ∼ 0.9, demonstrating that the majority of the available power can be focussed to a single point at the output. Several factors contribute to the fact that *p*_r_ < 1 even in the over-sampled case: the accuracy with which the required input field is generated with the DMD; any small drift of the optical system; the hard edge of the disk inside which power is considered in the focus; and the low-level camera noise—which, although low, is spread over many pixels compared to the size of the focus. Figure 2b, leftmost panel, shows a map of the power-ratio across the distal facet in this over-sampled case.

Figure 2c shows a graph of the mean power-ratio *p*_r_ as a function of the compression ratio *c* when our different reconstruction strategies are applied. We see that without the inclusion of any priors, *p*_r_ is once again linearly proportional to *c*, and thus, for low compression ratios, the contrast of the spots that can be created on the distal facet is low. This case is equivalent to partial TM measurement and has been previously considered in, for example, refs. ^9,38,39^. By incorporating prior knowledge and reconstructing the TM by solving Eq. 2, we move to a regime where *p*_r_ > *c*. As the compression ratio is reduced, *p*_r_ can significantly exceed *c* for an under-sampled measurement set. For example, using a sparsity prior alone yields a high-fidelity TM reconstruction; a mean power ratio of *p*_r_ > 0.8 is maintained down to a compression ratio of *c* ~ 0.2 in this case. This situation is further improved by incorporating the predicted support of the TM, which here yields a power-ratio approaching *p*_r_ = 0.9 when *c* ~ 0.1, corresponding to only 74 probe measurements. In this case, we estimate the level of mode coupling as $\sigma_{\ell}$ = 4 and *σ*_p_ = 2. SI Fig. S5 shows that at a compression ratio of *c* = 0.15, the reconstruction is relatively robust to variations in these support parameters. Figure 2d shows how the level of correlation between the under-sampled and fully sampled TMs varies as a function of *c*, which also demonstrates a similar trend.

While the power-ratio provides a useful measure of TM reconstruction fidelity that is directly related to scanning imaging, it is also insensitive to minor changes in the quality of the focus inside the small disk used to measure it. In contrast, the correlation curves are sensitive to any differences between the fully sampled and measured TMs. This explains the small differences in the shapes of the curves in Fig. 2c, d.

**Fig. 3:**
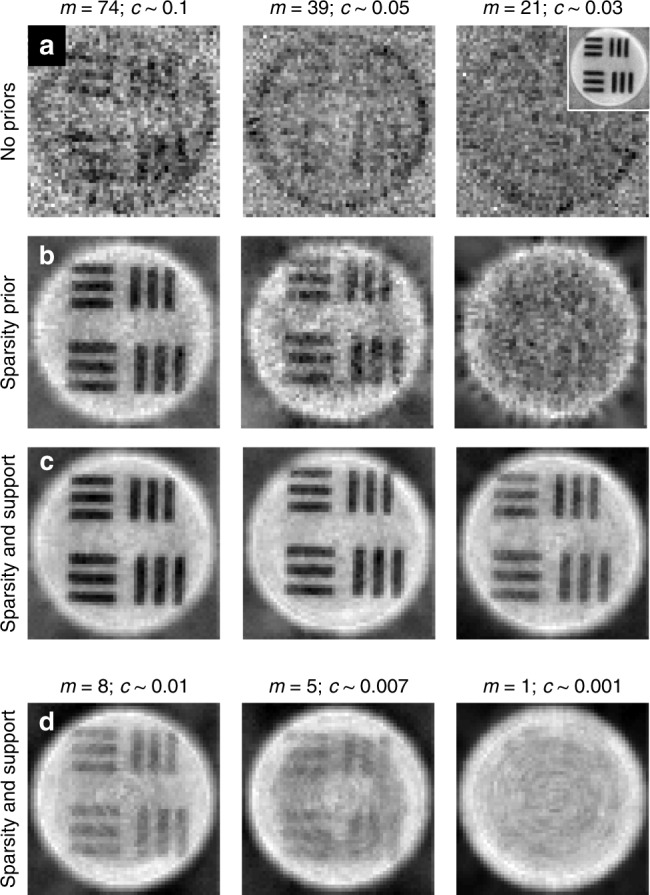
Imaging using compressively recovered TMs. **a** When using a TM reconstructed with no priors, at progressively decreasing levels of sampling (from left to right), imaging is practically impossible at these compression levels. Inset: image captured using a fully sampled TM. **b** Images obtained using a TM reconstructed with a sparsity prior. **c** Results obtained using sparsity and support priors. **d** Results with sparsity and support priors at further reduced levels of sampling. In this case, no image is discernible when attempting to infer the TM from a single measurement

We next directly tested the imaging performance of the compressively sampled TMs by imaging a resolution target positioned at the output facet of the MMF. Figure 3 shows transmission scanning images recorded by sweeping foci across the output facet that were generated using TMs reconstructed with the three different strategies; see the SI for details. We observe that without the use of priors, the images of the resolution target are barely discernible at a compression ratio of *c* ∼ 0.1 (Fig. 3a). Incorporating a sparsity constraint enables discernible imaging down to *c* ∼ 0.05 (Fig. 3b). The further inclusion of the support boosts the imaging contrast at *c* ∼ 0.05 and enables lower-contrast imaging down to *c* ∼ 0.01, corresponding to only eight probe measurements (Fig. 3c, d).

**Fig. 4:**
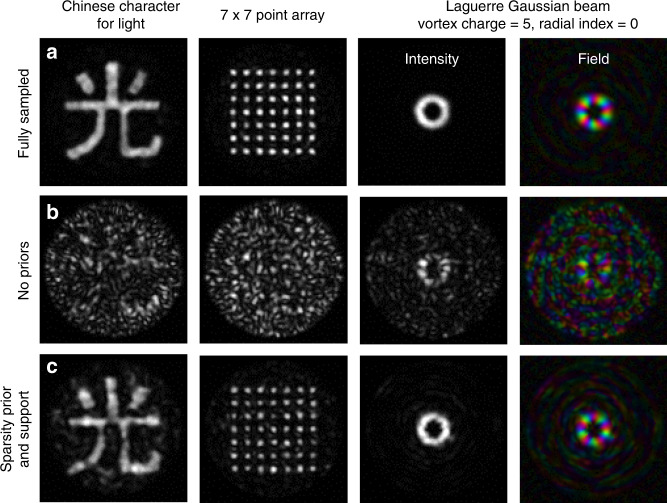
Pattern projection using compressively recovered TMs. **a** Results of using a fully sampled TM to project, from left to right, the Chinese character for light, a 7 × 7 point array, and a Laguerre–Gaussian (LG) beam carrying a vortex charge of $\ell$ = +5. The rightmost panels show the measured amplitude (brightness) and phase (colour) of the projected LG field reconstructed using digital holography^26^ by processing the interference pattern created by interference with a coherent plane-wave reference beam. The scale bar is the same as that used in Fig. 1. **b** When using an under-sampled TM (*c* = 0.2) without priors, it is not possible to project the target patterns. **c** When the TM is reconstructed using both a sparsity prior and an estimated support, the TM fidelity is sufficiently high to successfully project the target patterns

In addition to scanning imaging, accurate TM reconstructions also enable the projection of arbitrary patterns onto the distal facet. The projection of extended patterns is a more challenging test than the creation of focussed spots, as even small inaccuracies in the TM introduce strong speckling effects (i.e. extended patterns are more susceptible to perturbations). Figure 4 shows a comparison of the pattern projection capabilities of TMs reconstructed with full sampling (Fig. 4a), at a compression ratio of *c* ∼ 0.2 without priors (Fig. 4b), and using sparsity and support priors (Fig. 4c). We tested the system by generating the Chinese character for light, a 7 × 7 array of points, and a Laguerre–Gaussian beam. Evidently, at a compression ratio of *c* ∼ 0.2, it is virtually impossible to project patterns through the fibre without the use of priors when reconstructing the TM.

**Fig. 5:**
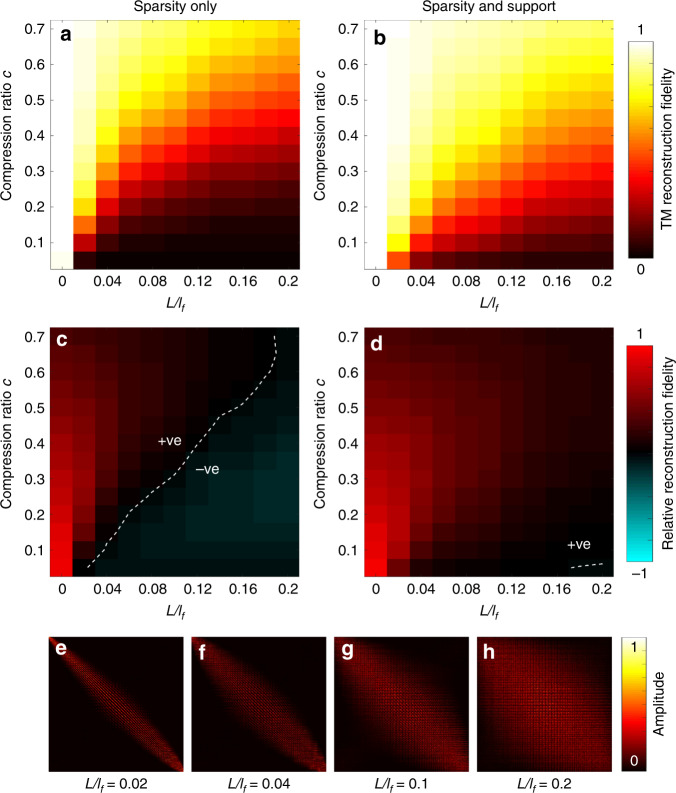
Simulated performance of compressive TM measurement for increasing levels of modal coupling. **a** A heat map showing the normalised TM reconstruction fidelity, when using a sparsity prior only, as a function of the degree of modal coupling (*L*/*l*_f_) and the compression ratio *c*. **b** Normalised TM reconstruction fidelity using sparsity and support priors. **c** The TM reconstruction fidelity depicted in (**a**) relative to the fidelity achievable using the columnwise method (which has a fidelity of *c*), i.e. the normalised TM reconstruction fidelity minus *c*. In the positive region, FISTA outperforms the columnwise method. In the negative region, the columnwise method outperforms FISTA. The white line represents the dividing line between the two regions. **d** The TM reconstruction fidelity shown in (**b**) relative to the columnwise method. **e**–**h** Examples of the absolute values of simulated TMs showing the tested range of modal coupling. **e** corresponds to a level of modal coupling similar to that observed in our experiment

Clearly, the compression ratios that may be achieved greatly depend on the strength of the available prior information. To investigate how the compression ratio scales with the level of modal coupling in a fibre, we conducted a series of numerical simulations, the results of which are shown in Fig. 5. We simulated a set of fibre TMs with increasing levels of modal coupling, quantified by *L*/*l*_f_, and assessed the performance of our reconstruction algorithms for a range of compression ratios. Figure 5a shows the fidelity of TM reconstruction using a sparsity prior only. Figure 5c shows the region of the parameter space in which compressive TM reconstruction outperforms the columnwise method. Figure 5b, d show the improvement achieved by also exploiting knowledge of the TM support. In this latter case, compressive reconstruction outperforms the columnwise method for compression ratios down to *c* = 0.05 for modal coupling up to *L*/*l*_f_ = 0.2. Figure 5e–h show examples of the absolute values of the simulated TMs over the coupling range studied. We note that for longer fibres, adopting a support that integrates prior knowledge of the anticipated levels of modal loss, in addition to modal coupling, may extend the range over which compressive TM reconstruction offers an advantage.

## Discussion

In this article, we have shown how the framework of compressive sensing can be employed to reduce the number of measurements required to reconstruct high-dimensional optical transmission matrices. Here, we have demonstrated this approach to measure the TM of an MMF, but the method is applicable to any scattering system for which we have access to some prior knowledge about the basis in which the TM is likely to be sparse. For example, diffusers, thin layers of biological tissue, and opaque walls all exhibit a tilt-tilt memory effect^21,23^ and thus have a quasi-diagonal TM in the real-space basis if the input and output planes are placed immediately adjacent to the scattering object itself (we note that if the planes are elsewhere, then as long as the distances from the scatterer to the chosen input and output planes are known, the input and output planes can be digitally transformed to planes adjacent to the object). Therefore, in these cases, compressive TM measurement can be achieved by performing an under-sampled set of Fourier basis probe measurements (i.e. plane waves incident from a range of different angles) and then iteratively reconstructing the TM while enforcing sparsity in the real-space basis. For a diffusive medium, the real-space sparsity pattern can be estimated, for example, by predicting the degree of lateral modal cross-talk based on the level of diffusion expected through a sample of a known transport mean free path and thickness. Interestingly, some thin anisotropically scattering samples exhibit quasi-diagonal TMs in both the real-space basis and the Fourier basis^22,40^. In this case, our algorithm could be extended to enforce sparsity in both of these bases—with the greater level of prior knowledge potentially leading to higher compression ratios.

In our proof-of-principle demonstration, we have assumed the following prior knowledge about the fibre under test: the manufacturer’s quoted values of the NA and core diameter and the approximate length. We do not need to know the bending configuration, as the majority of the observed modal cross-talk is due to the misalignment of the input and output^24^. Using a sparsity prior only, the level of compression we can expect is governed by *c* ∼ *s*log(*N*)^25^, where *s* is the level of sparsity of the TM, i.e. the proportion of the TM elements that contain appreciable power. As we have shown, the achievable compression level is further improved with additional information about the sparsity pattern. Here, we have focussed on short lengths (∼30 cm) of MMF, which have attracted increasing attention recently for applications in micro-endoscopy^28–30^ and in emerging classical and quantum optical computing schemes^19,20^. However, we envisage that our compressive sampling strategy may also be extended to longer segments of MMF, in which case higher levels of modal and polarisation coupling may reduce the extent of compression possible, as demonstrated in Fig. 5. We note that as long as an MMF is in the weak coupling regime, i.e. the power in the TM of the MMF is biased towards the main diagonal in the PIM basis, compressive sampling may still be leveraged. For example, ref. ^14^ has demonstrated that few-mode MMFs of up to 100 m in length are still in the weak coupling regime, with power found in predictable regions of the TM.

In our experiment, a key factor in accurately estimating the basis in which the TM is diagonal is alignment of the input to and output from the MMF. This alignment is non-trivial, as there are six degrees of freedom to consider at each end: the position of the objective lens with respect to the fibre in the *x*, *y* and *z* directions and the tip, tilt and defocus. Previous work has shown that a coarse indication of the level of misalignment of these degrees of freedom can be extracted from the fully sampled TM of the MMF itself and, hence, digitally corrected; see the SI of ref. ^24^. In this work, we have found that compressively sampled TMs, when represented in the real-space basis, also provide coarse information on the level of misalignment. Therefore, to accurately estimate the PIM basis, we manually aligned the experimental system (see SI Fig. S2) and performed an under-sampled set of measurements. We then analysed the raw data to extract coarse estimates of the input and output misalignments. These misalignments were then digitally corrected by absorbing them into the real-space-to-PIM matrices used to transform from the TM basis into the PIM basis for TM reconstruction to commence^23,24^.

The time taken to perform iterative TM reconstruction is also worth considering. In this work, the FISTA processing typically took ∼45 s; however, we note that there is a route to significantly reduce this optimisation time to <1 s (see the Methods). We also tested the performance of a faster reconstruction method based on Tikhonov regularisation that exhibited lower fidelity but took only ~4 s to complete (see SI Fig. S6). One area in which we expect the compressive sampling of TMs to be particularly advantageous is situations in which measurements cannot be made rapidly, for example, when using phase-only spatial light modulators of a relatively low modulation rate or in the case of a low SNR. Compressive sampling also has potential in higher-dimensional cases, such as the measurement of multispectral TMs, for which the number of measurements can run into the hundreds of thousands^41–44^.

We note that previous work has also implied the use of compressive TM recovery in some specific cases. For example, Gordon et al. recently showed that the TM of a fibre bundle can be recovered using fewer output images than the number of fibres by noting that fibres couple only to their neighbours (i.e. the TM is sparse in real space)^45^. Antipa et al. have demonstrated the measurement of the incoherent 3D intensity TM of a diffuser using a single image, under the assumption that the diffuser is a thin phase screen with an infinite memory effect^46^. Carpenter et al. have previously highlighted that the TM of a graded-index MMF can be approximately represented as a block-diagonal structure, which means it is necessary only to measure coupling within the blocks^47^. Our aim here is to highlight that compressive sampling may be applied to reconstruct TMs that are sparse in any known basis, with a number of camera frames that is lower than the mode capacity of the system. We have also shown how knowledge of the sparsity pattern can be leveraged and have presented the first experimental demonstration (to our knowledge) of compressively sampling the TM of an MMF using this technique.

Recently, machine learning (ML) approaches, such as deep neural networks, have been employed to learn the non-linear relationship between coherent fields projected onto one end of a fibre and the resulting speckle intensity patterns transmitted from the other end^48^. These methods have shown greater levels of robustness to small changes in fibre configuration than TM approaches, as they rely on a large number of measurements (typically many times more than the number of supported fibre modes) and thus incorporate information about the fibre in a range of states. However, we note that there is a key difference between the ML-based methods that have been demonstrated so far and TM-based approaches: knowledge of the TM permits scanning imaging of arbitrary scenes at the distal facet, while ML approaches, to maintain tractability, are typically trained to recognise only a subset of the possible coherent light fields at the distal facet—often with either the phase or the amplitude of the incident fields in the training data being held constant. This is because there are many possible fields at the distal facet that could result in the same intensity speckle pattern at the proximal facet, so the set of possible fields must be constrained in some way. Therefore, current ML-based methods are limited to imaging artificially synthesised light fields that possess the same general characteristics as the training set^49^. Nonetheless, ML techniques represent a novel approach and an intriguing avenue for further investigation. In this context, our compressive TM sampling strategy may have the potential to reduce the number of physical measurements that need to be performed and thus significantly speed up the training of ML algorithms. For example, a series of TMs could be rapidly compressively acquired as a fibre moves through a number of different configurations^50^. This set of TMs could then be used to numerically simulate large collections of ML training data much more quickly than they could be measured experimentally^51^.

Finally, we highlight that the concept of compressive TM reconstruction may be interpreted as constrained phase retrieval in a large number of dimensions. In phase retrieval, the objective is typically to estimate unknown phase components of a complex field with access only to the intensity of the field and some constraints^52,53^. Here, we have access to both the intensity and phase of an under-sampled set of measurements that are linked through a high-dimensional linear system of equations (i.e. the TM), and the iterative approach we have used to solve this problem is similar to those used in phase retrieval problems^54,55^. More broadly, the concept of compressive sensing has been combined with the high-dimensional transformations enacted by scattering systems in several other ways in the past. Most notably, compressive sensing has been applied to reduce the number of measurements required to recover images through scattering systems by drawing on priors relating to the form of the images^56–58^. Our work complements these previous studies by highlighting that it is also possible to draw on priors concerning the *scatterer itself* during the calibration phase. In the future, we hope that the concept of compressive TM reconstruction can be combined with the ultra-fast modulators currently under development^59,60^, unlocking the potential to characterise and image objects through even dynamically changing scattering systems as efficiently as possible.

## Materials and methods

### Constructing the sensing matrix

Consider an MMF with a mode capacity of *N* modes per polarisation, with an unknown TM **T** ∈ C^*N* × *N*^, represented in the PIM basis. In our work, the PIM basis is the natural choice, as it is the basis in which the sparsity priors are enforced.

To fully sample the *N* × *N*-element TM, we inject *N* orthogonal probe modes, **a**_1_, **a**_2_,…, **a**_*N*_ ∈ C^*N*^, also expressed in the PIM basis. The transformation of these inputs by the MMF produces the following outputs:

$$\begin{array}{l}{\mathbf{b}}_1 \,=\, {\mathbf{Ta}}_1\\ {\mathbf{b}}_2 \,=\, {\mathbf{Ta}}_2\\ \,\,\,\,\,\,\,\, \vdots \\ {\mathbf{b}}_N \,=\, {\mathbf{Ta}}_{\it{N}}\end{array}$$

Horizontally concatenating the corresponding sides of each of the above equations and taking the transpose of the resulting matrix, we obtain the following matrix product:

3 $$\begin{array}{l}\left[ {{\mathbf{b}}_1,\,{\mathbf{b}}_2,\, \ldots ,\,{\mathbf{b}}_N} \right]^{\mathrm{T}} \,=\, \left[ {{\mathbf{Ta}}_1,\,{\mathbf{Ta}}_2,\, \ldots ,\,{\mathbf{Ta}}_N} \right]^{\mathrm{T}}\\ \quad \quad \quad \quad \quad \quad \,\, \,=\, \left( {{\mathbf{T}}\left[ {{\mathbf{a}}_1,\,{\mathbf{a}}_2, \ldots ,\,{\mathbf{a}}_N} \right]} \right)^{\mathrm{T}}\\ \quad \quad \quad \quad \quad \quad \,\, \,=\, \left[ {{\mathbf{a}}_1,\,{\mathbf{a}}_2,\, \ldots ,\,{\mathbf{a}}_N} \right]^{\mathrm{T}}{\mathbf{T}}^{{\mathrm{T}}}\\ \quad \quad \quad \quad \quad \quad \,\, \,=\, {\mathbf{AT}}^{{\mathrm{T}}}\end{array}$$

where (·)^T^ denotes the matrix transpose operator. Vectorising both sides of the above equation gives an equivalent matrix-vector form:

4 $$\begin{array}{l}{\mathrm{vec}}\left( {\left[ {{\mathbf{b}}_1,\,{\mathbf{b}}_2,\, \ldots ,\,{\mathbf{b}}_N} \right]^{\mathrm{T}}} \right) \,=\, {\mathrm{vec}}\left( {{\mathbf{AT}}^{\mathrm{T}}} \right)\\ \quad \quad \quad \quad \quad \quad \quad \quad \,\,\,\,\, \,=\, \left( {{\mathbf{I}}_N \,\otimes\, {\mathbf{A}}} \right)\,{\mathrm{vec}}\left( {{\mathbf{T}}^{\mathrm{T}}} \right)\end{array}$$

where vec(·) denotes the vectorisation operator, **I**_*N*_ is the *N* × *N* identity matrix, and the symbol $\otimes$ denotes the Kronecker matrix product between two matrices. The last equality in Eqn. 4 follows from the vectorisation-Kronecker product identity: vec(**PXQ**) = (**Q**^T^ $\otimes$ **P**) vec(**X**). Finally, by letting:

$$\begin{array}{l}{\mathbf{y}}\,\mathop { = }\limits^{{\mathrm{def}}}\, {\mathrm{vec}}\left( {\left[ {{\mathbf{b}}_1,\,{\mathbf{b}}_2,\, \ldots ,\,{\mathbf{b}}_N} \right]^{\mathrm{T}}} \right)\\ \,\,\,{\mathbf{S}}\,\mathop { = }\limits^{{\mathrm{def}}}\, \left( {{\mathbf{I}}_N \,\otimes\, {\mathbf{A}}} \right),\,{\mathrm{and}}\\ \quad \,\,{\mathbf{t}}\,\mathop { = }\limits^{{\mathrm{def}}}\, {\mathrm{vec}}\left( {{\mathbf{T}}^{\mathrm{T}}} \right)\end{array}$$

we obtain the desired form given in Eq. 1.

Since **S** = (**I**_*N*_ $\otimes$ **A**), it is an *N*^2^ × *N*^2^-block-diagonal matrix, with **A** repeated along the diagonal. Each row of **A** corresponds to a single probe mode, here expressed in the PIM basis. Therefore, reducing the number of measurements is equivalent to reducing the number of rows in **A** and, consequently, to reducing the numbers of rows in **S** and elements in **y**. When the TM is under-sampled, **S** has fewer rows than columns. Therefore, the application of priors within the framework of compressive sensing is necessary to uniquely solve Eq. 1. We also note that the block-diagonal nature of **S** means that the reconstruction of each column of the TM can be carried out independently and in parallel, facilitating rapid TM reconstruction if necessary.

### Estimating the support

To construct the *n*th column of the predicted TM support, we numerically define a discretised 2D Gaussian function *f* in ($\ell$, *p*)-space within the bounds of the power spectrum grid representing the indices of the allowed PIMs (see Fig. 2b):

5 $$f\left( \ell,p \right) \,=\, \exp \left[ { - \frac{{\left( {\ell \,-\, \ell _0} \right)^2}}{{2\sigma _\ell ^2}} \,-\, \frac{{\left( {p \,-\, p_{\mathrm{0}}} \right)^2}}{{2\sigma _{\mathrm{p}}^2}}} \right]$$

Centred on the nth PIM corresponding to indices ${\ell_0}$ and *p*_0_. Here, $\sigma_{\ell}$ and *σ*_p_ are standard deviations representing our estimated level of coupling. This 2D function is then reshaped into a column vector to form the *n*th column of the predicted TM support. This process is repeated while moving the centre of the Gaussian function over each PIM index to build up the entire predicted support, such as that shown in Fig. 1. The ordering of the PIMs of indices $\ell$ and *p* into a 1D list is arbitrary but must be self-consistent. Here, we follow the ordering used in refs. ^23,24^. Finally, the predicted support is vectorised to generate the **w** that is used in Eq. 2.

### Design of the hyper-uniform input basis

To ensure that the input facet is approximately evenly sampled, the locations of *m* points are selected by creating a hyper-uniform array. We first randomly distribute the m points across a disk representing the core of the fibre. To prevent the clustering that naturally occurs when the locations are randomly chosen, we iteratively update the positions of the points to evenly spread them across the core. This is achieved by defining a repulsive ‘force’ acting along the line that joins two points, the magnitude of which is inversely proportional to the distance between the points. The total resulting force vector acting on an individual point is the vector sum of the repulsive forces from all nearby points. In each iteration, we move each point in the direction of the total force vector acting on it. The size of the movement is a small distance (on the order of one one-hundredth of the core radius) proportional to the magnitude of the total force acting on each point. An additional force pointing radially inward is applied to points near the edge of the core to prevent the points from repelling each other beyond the radius of the core. The positions of the points are updated until no appreciable changes are observed. The resulting set of points then specifies the locations of the foci of the under-sampled input probe measurements.

### Solving the optimisation problem

Algorithm 1 describes the fast iterative soft-thresholding steps used to solve the problem in Eq. 2. This is also known as an *accelerated proximal gradient descent* algorithm. We implemented Algorithm 1 in MATLAB. *λ* was manually tuned once by testing the reconstruction performance for a range of choices for *λ* before choosing *λ* = 0.25. This value of *λ* was used for all reconstructions, irrespective of the compression ratio. The Lipschitz constant used as a bound for the choice of the step size was calculated by computing the singular value decomposition of the sensing matrix, which took ∼35 s at the outset. Alternatively, a simple back-tracking scheme could be used to perform automatic step size selection. **t**^0^ was initialised using the solution obtained from the columnwise method; for an example, see Fig. 1e. On a laptop with a quad core (8 threads) Intel i7-8565U CPU and 8 GB of RAM, it typically took ∼45 s to solve Eq. 1 with a compression ratio of *c* ∼ 0.15 at a fixed step size.

In Algorithm 1, the number of multiplications per iteration scales as the number of non-zero elements of the sensing matrix, which is equal to *mN*^2^. The time taken to perform these matrix multiplications represents the major time-limiting factor in the algorithm. Therefore, when the problem is solved as a single matrix equation, the time per iteration, *t*, increases as *t* ∝ *mN*^2^. In the present case, the reconstruction time is longer than the time taken to record the fully sampled TM using a fast DMD and a high-speed camera (∼10 s, excluding the pattern loading time). However, we note that this reconstruction time could be significantly reduced by specifically tailoring the optimisation algorithm to take advantage of the structure of the sampling matrix **S**. Here, we treated Eq. 2 as a single sparse matrix equation, but as mentioned above, the block-diagonal structure of **S** means that Eq. 2 is separable into, in this case, *N* = 754 smaller equations that can, in principle, be solved in parallel to recover each column of the TM independently. In this case, the number of multiplications required for a single iteration of each of these individual matrix equations is *mN*, and thus, the time per iteration for these equations also scales as *t* ∝ *mN*. We estimate that for our present case, for *N* = 754, this would reduce the reconstruction time to less than a second. We note that for larger *N*, the TM and the predicted support become sparser, and therefore, we expect the compression ratio to improve as *N* increases, i.e. the fraction of probe measurements required, *m*/*N*, will decrease with increasing *N*.

### Estimation of the transport mean free path in the PIM basis

The level of off-diagonal coupling in the fibre TM, when represented in the PIM basis, can be quantified by the ratio *L*/*l*_f_, where *L* is the fibre length and *l*_f_ is the transport mean free path in the PIM basis, i.e. the length of the fibre beyond which an arbitrary input mode statistically couples to all output modes. The fibre TM is considered fully coupled when *L*/*l*_f_ > 1.

We estimated *l*_f_ by numerically simulating the TMs of many short sections of fibre, each possessing a small degree of coupling, generated by randomly misaligning the inputs and outputs to/from each segment. We then modelled these sections of fibre connected end to end by calculating the product of their TMs. As more sections were included, the level of coupling in the TM increased, representing the ensemble of connected fibre sections. We numerically quantified the level of off-diagonal power in this TM by calculating *I*_w_, the weighted sum of the normalised power in the TM **T**, with each element weighted by its perpendicular distance to the diagonal:

6 $$I_{\rm{w}} \,=\, \frac{1}{{\sqrt 2 }}\mathop {\sum}\limits_{i \,=\, 1}^N {\mathop {\sum}\limits_{j \,=\, 1}^N {T_{i,j}T_{i,j}^ \ast |i \,-\, j|} }$$

where *i* and *j* index the rows and columns, respectively; *T*_*i*,*j*_ refers to the element in row *i* and column *j* of **T**; and **T**^∗^ is the complex conjugate of **T**. In each case, the TM was normalised such that:

7 $$\mathop {\sum}\limits_{i \,=\, 1}^N {\mathop {\sum}\limits_{j \,=\, 1}^N {T_{i,j}T_{i,j}^ \ast \,=\, 1} }$$

We plotted *I*_w_ as a function of the fibre length. *I*_w_ initially increases as the power spreads gradually farther from the diagonal and then plateaus once *L* = *l*_f_, enabling *l*_f_ to be estimated. We could also use this model to estimate the value of *L*/*l*_f_ observed in our experiment by finding the length of fibre in our model that would result in the same degree of coupling observed in our experiment.

While this model provides a way to estimate *l*_f_ and to quantify the level of coupling in the TM, we note that the way this model scales with *L* assumes that the coupling is solely due to the mixing of light during propagation through the fibre and under-estimates the cross-talk due to misalignment of the input and output. In our experiment, a major source of the observed coupling was the misalignment of the light input to and output from the fibre.

## Supplementary information

Supplementary Information

Movie 1
