# Supplementary material for: Compressively sampling the optical transmission matrix of a multimode fibre
Source: Light Sci Appl. 2021 Apr 21;10:88. doi: 10.1038/s41377-021-00514-9 (PMC8060322; doi:10.1038/s41377-021-00514-9)
Supplement: Supplementary file 1 — Supplementary Information [file 41377_2021_514_MOESM1_ESM.pdf]

Compressively sampling the optical transmission matrix of a multimode fibre: supplementary information

Shuhui Li,[1,2,*] Charles Saunders,[3] Daniel J. Lum,[4] John Murray-Bruce,[3,5]
Vivek K Goyal,[3] Tomáš Čižmár,[6,7] and David B. Phillips[1,†]

[1]Physics and Astronomy, University of Exeter, Exeter, EX4 4QL. UK.
[2]Wuhan National Laboratory for Optoelectronics, School of Optical and Electronic Information, Huazhong University of Science and Technology, Wuhan 430074, Hubei, China.
[3]Department of Electrical and Computer Engineering, Boston University, Boston, MA, 02215, USA
[4]Department of Physics and Astronomy, University of Rochester, 500 Wilson Blvd, Rochester, NY 14618, USA
[5]Department of Computer Science and Engineering, University of South Florida, Tampa, FL, 33620, USA
[6]Leibniz Institute of Photonic Technology, Albert-Einstein-Straße 9, 07745 Jena, Germany
[7]Institute of Scientific Instruments of CAS, Královopolská 147, 612 64, Brno, Czech Republic

S1 Transformation matrix from spots to PIMs.
S2 Schematic of the experimental set-up.
S3 Fully-sampled TM in PIM basis reconstructed using the column-wise method.
S4 Under-sampled TM in PIM basis reconstructed using FISTA with sparsity and support priors.
S5 Robustness of reconstruction to inaccurate estimation of σ_ℓ and σ_p.
S6 Tikhonov regularisation results.
S7 Movie 1: Experimentally measured coupling of PIMs.

---

[1] Tomáš Čižmár, Michael Mazilu, and Kishan Dholakia. In situ wavefront correction and its application to micromanipulation. *Nature Photonics*, 4(6):388, 2010.
[2] Tomáš Čižmár and Kishan Dholakia. Exploiting multimode waveguides for pure fibre-based imaging. *Nature communications*, 3:1027, 2012.
[3] David B Phillips, Ming-Jie Sun, Jonathan M Taylor, Matthew P Edgar, Stephen M Barnett, Graham M Gibson, and Miles J Padgett. Adaptive foveated single-pixel imaging with dynamic supersampling. *Science advances*, 3(4):e1601782, 2017.

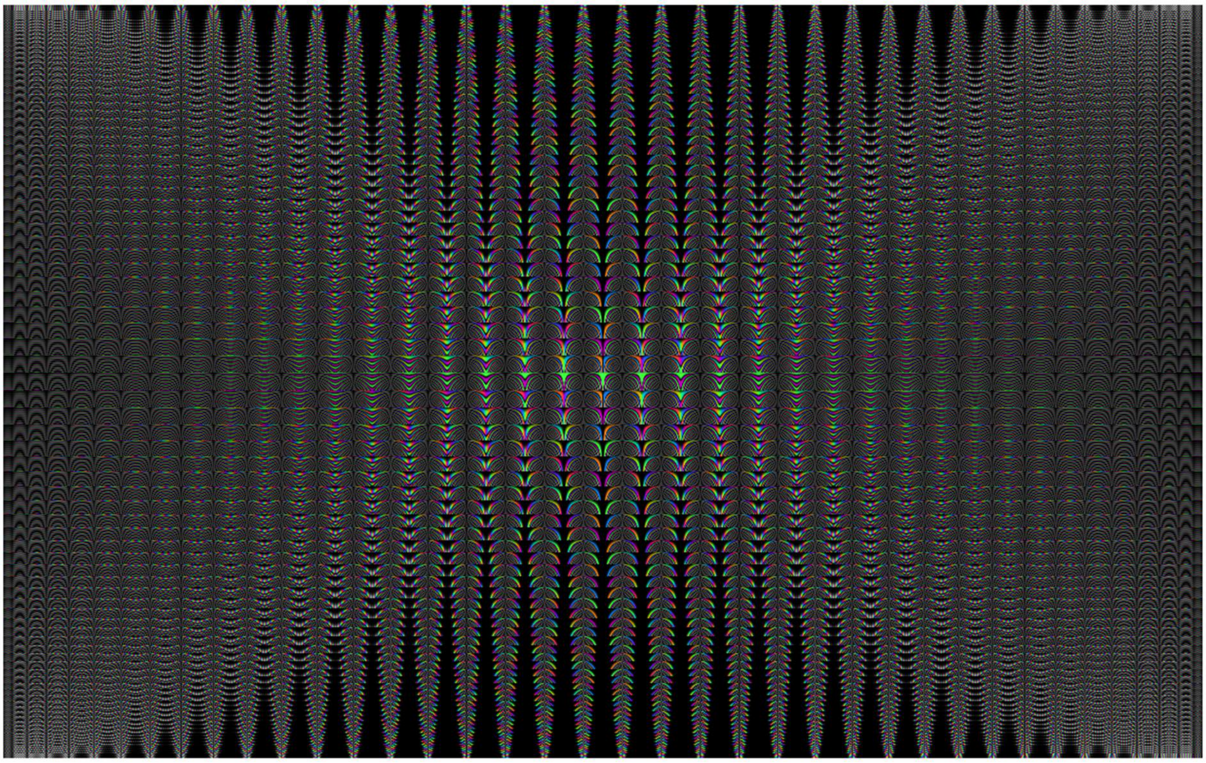

FIG. S1: **Transformation matrix from spots to PIMs**: Here columns represent spot locations, and rows represent PIMs. Therefore the pixel at row i, col. j represents the amplitude (brightness) and phase (colour) of the overlap between a spot focussed at location j on the input facet, with PIM i. We see that the majority of spot locations excite many PIMs – showing that the spot input basis is relatively incoherent with the PIM basis as required in our compressive sampling protocol.

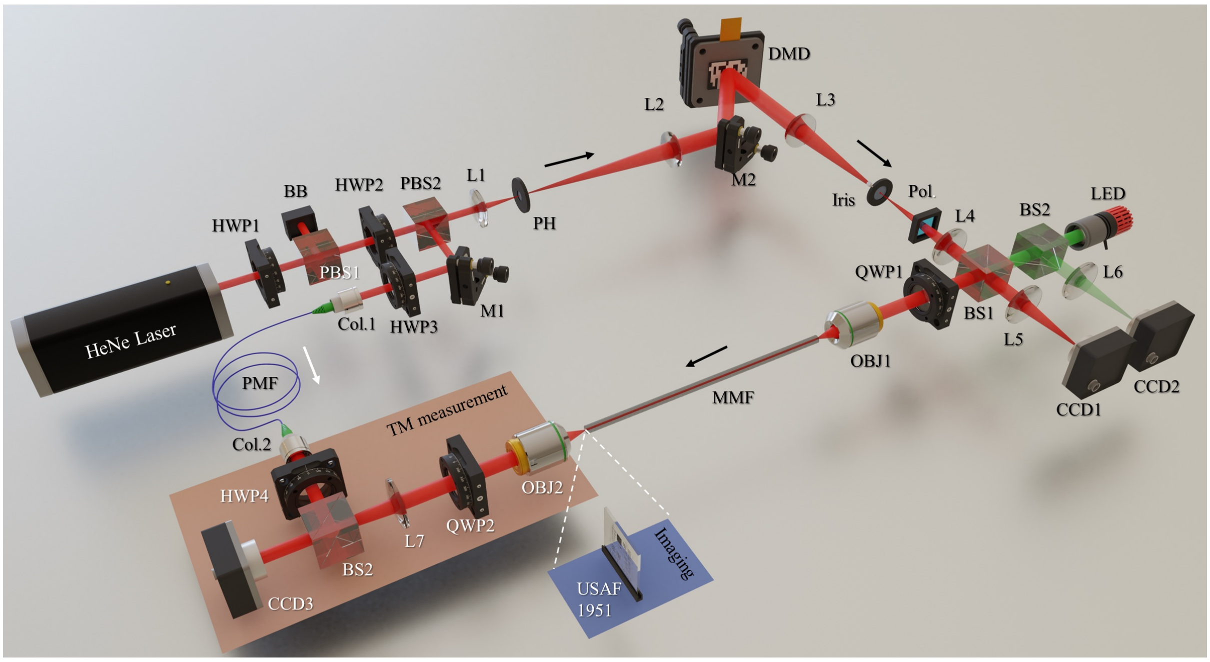

FIG. S2: **Schematic of the experimental set-up**: The optical system is based on a Mach-Zehnder interferometer. A laser beam is generated by a 1 mW Helium-Neon laser source operating at a wavelength of 633 nm. The beam is split into target and reference arms using a polarising beamsplitter (PBS2). In the target arm, the beam is spatially filtered and expanded to fill a digital micro-mirror device (DMD) (ViALUX V-7001). The first diffraction order of the DMD is selected by an iris which blocks all other diffraction orders. The DMD chip plane is imaged onto the pupil of an objective lens (OBJ1, 20X magnification). The input facet of the MMF is placed at the front focal plane of the objective lens. The MMF output facet is imaged onto a high-speed camera (CCD3, Basler Pilot GigE, resolution 648x488), where it interferes with the coherent reference beam. The plane wave of the reference beam arrives at the camera at a small tilt angle with respect to the camera chip normal, enabling single-shot digital holography to reconstruct the intensity and phase of the target field. CCD1 and CCD2 are alignment cameras (also Basler Pilot GigE). They are in the image plane of the proximal facet of the MMF. CCD1 images the incident laser beam, enabling aberration correction of the part of the optical set-up before the MMF if necessary, using, for example, the methods described in ref. [**?**]. This correction need only take place once and is unchanged regardless of the test scattering object that is placed in the TM measurement system. A red LED illuminator is used to illuminate the proximal facet of the fibre to aid alignment. Transmission imaging was achieved by scanning a focussed beam over a transmissive resolution target placed $\sim 40\mu$m from the distal facet of the fibre. The TM under question was used to calculate the input field required to generate a focussed spot on the output distal facet of the MMF. This was then refocussed from the distal fibre facet to the plane of the resolution target by adding a quadratic Fresnel lens phase function to the hologram displayed on the DMD, as described in ref. [**?**]. To reconstruct an image, the total transmitted intensity arriving at CCD3 was recorded for each spot location at the distal facet. Reflection imaging is also possible (as would be necessary for the MMF to be deployed in a real application), but in our case the low power of the laser used for the experiment, coupled with low collection efficiency for MMFs meant the returning signals were small, which introduced additional noise and so we did not use reflection images to test the performance of the reconstructed TMs.

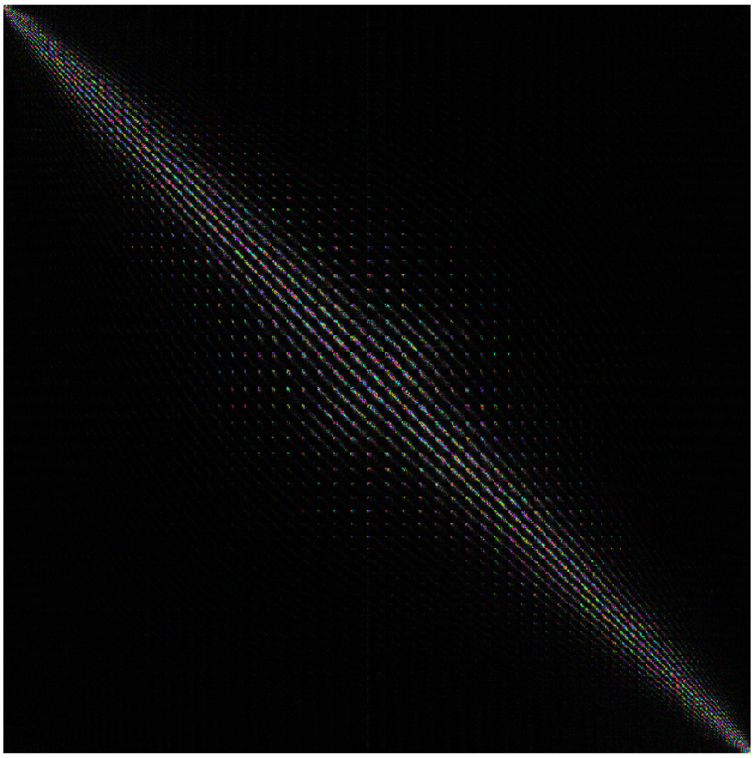

FIG. S3: **Fully-sampled TM in PIM basis reconstructed using the column-wise method**: This is a larger scale reproduction of main text Fig. 1(a). The TM is over-sampled using $c = 2.2$ as explained in the main text.

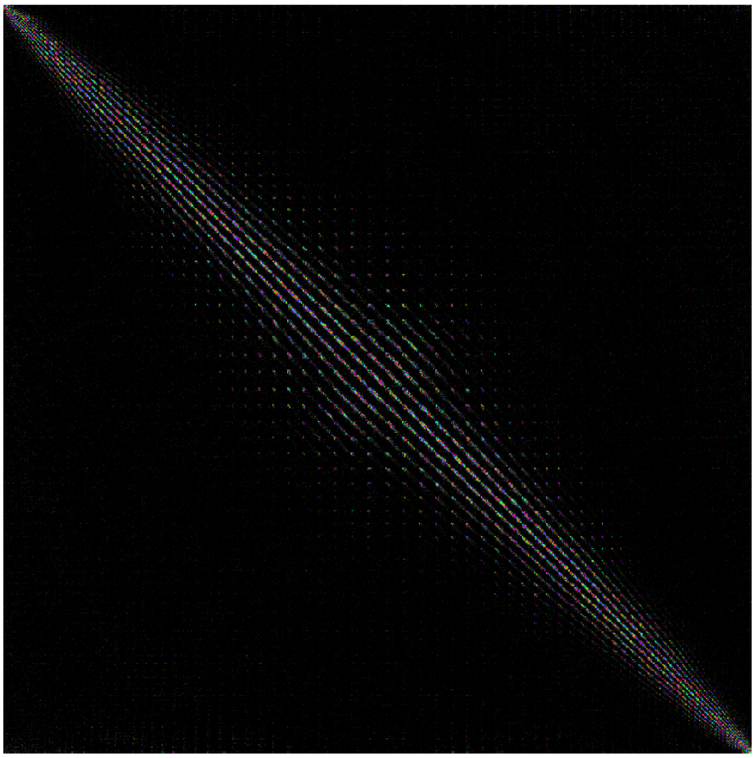

FIG. S4: **Under-sampled TM in PIM basis reconstructed using FISTA with sparsity and support priors**: This is a larger scale reproduction of main text Fig. 1(f). The TM is under-sampled using $c = 0.25$. The correlation with the fully sampled TM is 0.88.

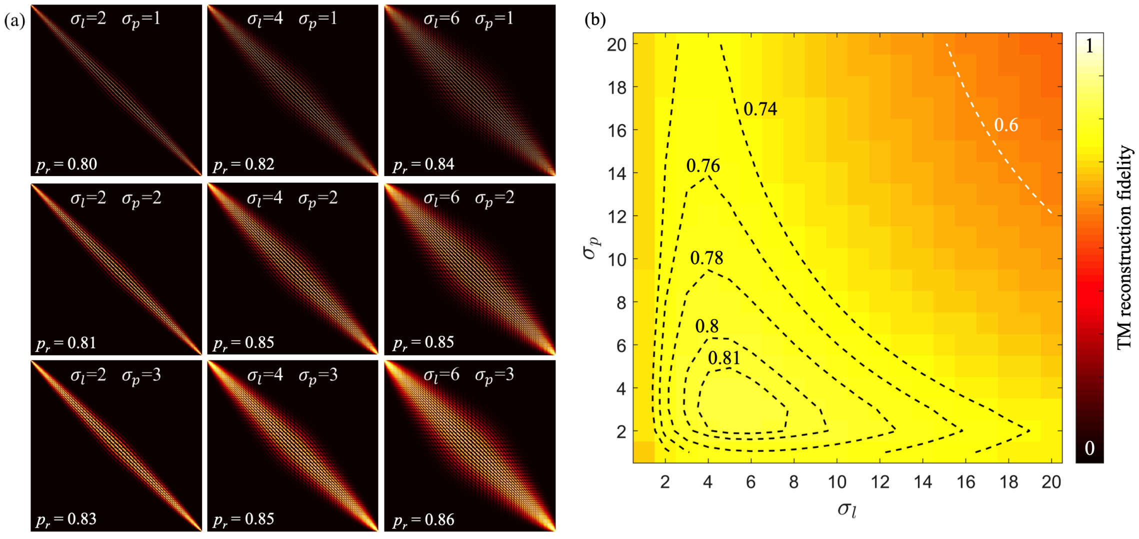

FIG. S5: **Reconstruction with variation in the estimated support**: **(a)** Here we experimentally reconstruct a TM with a compression ratio of $c = 0.15$ and investigate the variation in reconstruction fidelity when the parameters of the estimated support are varied. We trial nine different estimated supports, with $\sigma_\ell = 2 : 2 : 6$, and $\sigma_p = 1 : 1 : 3$. The mean power-ratio of foci generated at the output using TMs reconstructed with the nine different supports is annotated. In this case we find that even when the extent of the anticipated off-diagonal power coupling in the TM is over-estimated or under-estimated, the fidelity of the reconstructed TM is always higher than without using a support: $p_r > 0.8$ in every case tested here. This illustrates that the FISTA reconstruction is robust to inaccurate estimates of the support within the demonstrated range. **(b)** Here we simulate the TM reconstruction fidelity over a wider range of support parameters for the same compression ratio as (a). We find that the reconstruction can tolerate a significant overestimation in mask parameters in this case, with only gradual reduction in reconstruction fidelity. The white contour at 0.6 indicates the fidelity of the reconstruction using sparsity priors only.

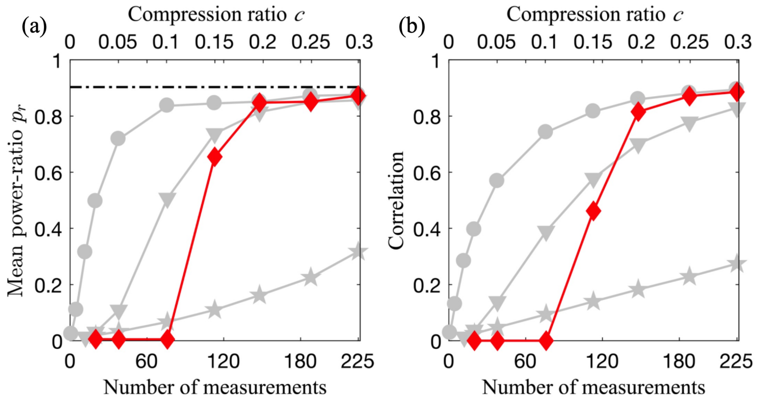

FIG. S6: **TM reconstruction using Tikhonov regularisation**: In addition to the three reconstruction methods presented in the main paper, we also investigated a reconstruction method based on Tikhonov regularisation. In this case the estimated TM amplitude support was thresholded to create a binary mask: containing 0 in regions in which we expect there to be minimal power in the reconstructed TM, and 1 otherwise. To promote solutions that have low absolute values in regions specified by the predicted mask, the information about which regions of the TM we expect to be zero can be inserted into Eqn. 1 (main text), by adding extra rows to $\mathbf{S}$ and $\mathbf{y}$. For example, to specify that the nth entry in $\mathbf{t}$ is 0, we vertically concatenate $\mathbf{S}$ with an extra row consisting of all elements set to 0 except for the nth element which is set to 1. We also vertically concatenate $\mathbf{y}$ with an extra element set to 0. This can be repeated for every element of the TM that we expect to be 0 according to the predicted binary mask. Note that the memory requirements are low for this approach as the extra rows are mainly zeros and can be represented using sparse matrices. As long as enough additional information has been inserted into Eqn. 1 (main text) to render it full-rank, then it can be solved using standard fast methods that minimise an error term η given by the square of the Euclidean norm of the residual: $\eta = \|\mathbf{St} - \mathbf{y}\|_2^2$, which attempts to account for any inconsistencies due to inaccuracies in the estimation of the support or noise in the measurements. Additionally, the strength of the predicted support priors can be weighted with respect to the probe measurements by a factor λ_{Tik}, for example, using the methods described in the SI of ref. [**?**]. This method is a form of Tikhonov regularisation: to demonstrate this, let $\mathbf{\Gamma}$ represent the matrix formed from the extra rows vertically concatenated with $\mathbf{S}$ as described above, and $\mathbf{0}$ is a column vector representing the extra rows of zeros vertically concatenated with $\mathbf{y}$. Therefore the new matrix-vector equation becomes: $\begin{bmatrix} \mathbf{S} \\ \lambda_{\mathrm{Tik}}^{1/2}\mathbf{\Gamma} \end{bmatrix} \mathbf{t} = \begin{bmatrix} \mathbf{y} \\ \mathbf{0} \end{bmatrix}$, where $\lambda_{\mathrm{Tik}}^{1/2}$ is the weighting factor. In this case the square of the Euclidean norm of the residual is given by $\left\| \begin{bmatrix} \mathbf{S} \\ \lambda_{\mathrm{Tik}}^{1/2}\mathbf{\Gamma} \end{bmatrix} \mathbf{t} - \begin{bmatrix} \mathbf{y} \\ \mathbf{0} \end{bmatrix} \right\|_2^2 = \left\| \begin{bmatrix} \mathbf{St} - \mathbf{y} \\ \lambda_{\mathrm{Tik}}^{1/2}\mathbf{\Gamma t} \end{bmatrix} \right\|_2^2 = \|\mathbf{St} - \mathbf{y}\|_2^2 + \lambda_{\mathrm{Tik}} \|\mathbf{\Gamma t}\|_2^2$, which is equivalent to Tikhonov regularisation with a Tikhonov matrix $\mathbf{\Gamma}$ and weighting factor λ_{Tik}. Here we set $\lambda_{\mathrm{Tik}} = 1$. **(a)** and **(b)** show the performance of Tikhonov regularisation (diamonds) in comparison with the other three reconstruction strategies (data shown in grey equivalent that in main text Figs.2(c,d)). In this case for compression ratios of $c > 0.25$, the performance of Tikhonov regularisation is equivalent to FISTA using sparsity and support priors, but the solution is returned approximately an order of magnitude faster. However, the solution is sensitive to under-estimations of the level of off-diagonal power spread in the TM. Additionally, for lower compression ratios of $c < 0.2$, the above matrix equation is not full-rank, and since no sparsity priors are invoked the Tikhonov reconstruction undergoes catastrophic failure: the number of probe measurements must be large enough to render the matrix equation full rank for an accurate solution to be found. In comparison, all other reconstruction strategies undergo graceful failure as c is reduced. Therefore, although relatively fast to perform, Tikhonov reconstruction can only be successfully applied for mild compression ratios in circumstances where the level of off-diagonal power spread can be safely over-estimated.

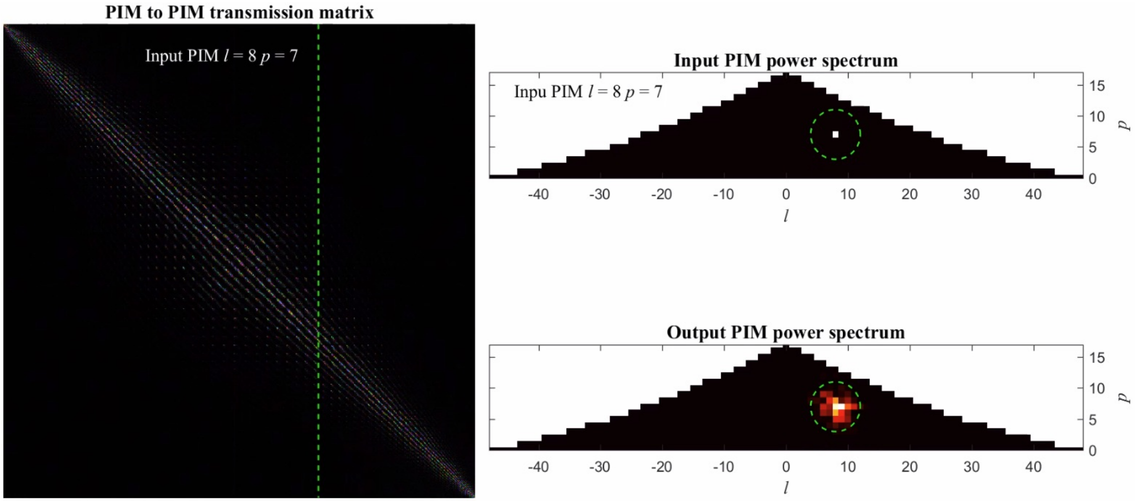

FIG. S7: **Movie 1: Experimentally measured coupling of PIMs**: The figure depicts a frame from supplementary movie 1 showing the level of coupling each input PIM undergoes due to propagation through the MMF. The left hand panel shows the measured TM. The vertical green line highlights a single TM column. The nth column corresponds to the nth input mode. Each row captures the degree of coupling with all other PIMs experienced by the nth input PIM after propagating through the MMF. The upper right hand plot highlights the nth input PIM in (ℓ,p)-space with a bright point. The lower right hand plot shows the level of power coupling of the nth PIM at the fibre output. We see that power couples locally in (ℓ,p)-space. We exploit this to predict the support of the TM. We also note that PIMs with low p-indices tend to couple more broadly to the neighbouring PIMs, as the values of the phase velocities of these PIMs are closer. There is potential for this observation to be exploited as more detailed prior knowledge about the structure of the TM in the future.
